# *MED23* pathogenic variant: genomic–phenotypic analysis

**DOI:** 10.25122/jml-2024-0065

**Published:** 2024-05

**Authors:** Ahmed Bamaga, Osama Muthaffar, Anas Alyazidi, Sarah Bahowarth, Mohammed Shawli, Fahad Alotibi, Matar Alsehemi, Mohammad Almohammal, Adel Alawwadh, Njood Alghamdi

**Affiliations:** 1 Department of Pediatrics, Faculty of Medicine, King Abdulaziz University, Jeddah, Saudi Arabia; 2 Neuromuscular Medicine Unit, Department of Pediatrics, Faculty of Medicine, King Abdulaziz University Hospital, Jeddah, Saudi Arabia; 3 Faculty of Medicine, King Abdulaziz University, Jeddah, Saudi Arabia; 4 Pediatric Neurology Unit, Department of Pediatrics, King Fahad Hospital, Albaha, Saudi Arabia; 5 Department of Pediatrics, Ministry of Health, Bisha, Saudi Arabia; 6 Department of Pediatrics, Khamis Mushait Maternity and Children Hospital, Abha, Saudi Arabia; 7 Faculty of Medicine, Albaha University, Albaha, Saudi Arabia

**Keywords:** *MED23*, ID, refractory epilepsy, developmental delay, ketogenic diet

## Abstract

The mediator complex subunit 23 (*MED23*) gene encodes a protein that acts as a tail module mediator complex, a multi-subunit co-activator involved in several cellular activities. *MED23* has been shown to have substantial roles in myogenesis and other molecular mechanisms. The functions of *MED23* in the neurological system remain unclear and the clinical phenotype is not thoroughly described. Whole exome sequencing was used to identify a novel mutation in the *MED23* gene. DNA capture probes using next-generation sequencing-based copy number variation analysis with Illumina array were performed. The clinical, demographic, neuroimaging, and electrophysiological data of the patients were collected, and similarly, the data of all reported cases in the literature were extracted to compare findings. Screening a total of 9,662 articles, we identified 22 main regulatory processes for the *MED23* gene, including suppressive activity for carcinogenic processes. *MED23* is also involved in the brain’s neurogenesis and functions. The identified cases mainly presented with intellectual disability (87.5%) and developmental delay (50%). Seizures were present in only 18.75% of the patients. Slow backgrounds and spike and sharp-wave complexes were reported on the electroencephalogram (EEG) of a few patients and delayed myelination, thin corpus callosum, and pontine hypoplasia on magnetic resonance imaging (MRI). The *MED23* gene regulates several processes in which its understanding promotes considerable therapeutic potential for patients. It is crucial to consider genetic and laboratory testing, particularly when encountering potential carriers. Intellectual disability and developmental delay are the most notable clinical signs with heterogeneous features on EEG and MRI.

## INTRODUCTION

As a component of the core transcriptional machinery, the mediator complex sub-unit 23 gene (*MED23*; OMIM# 605042) encodes a protein that acts as a tail module mediator complex and a multi-subunit co-activator involved in several cellular activities [1,2]. These mutations cause the deregulation of important genes that are required for early brain development, affecting normal child growth [3]. The majority of the time, pathogenic alterations are thought to impact the loss-of-function of *MED23* function by affecting the clustering of residues in N-HEAT, 3-HEAT, and 5-HEAT.

Recently, four individuals with intellectual disabilities (IDs) were reported to have it [3,4]. Over the following two decades, several other mutant *MED23* alleles linked to complex III deficiency have been reported and associated with a set of mitochondrial disorders of varying severity, ranging from early onset, lethal diseases, to mild conditions with chronic clinical courses [5–7]. In addition to these biological processes, *MED23* has been shown to have substantial roles in myogenesis, lung carcinogenesis, glucose and lipid metabolism, T cell activation, enhanced neural differentiation, and osteoblast differentiation [8–14]. The functions of *MED23* in the neurological system remain unclear. According to descriptions of further cases, the phenotype may be more complicated depending on the kind, location, and effect of *MED23* mutations [4,15–17], whereas the ‘classical phenotype’ is defined as having characteristics such as microcephaly, axial hypotonia, epilepsy, dystonia, and spasticity. Additional features such as screaming spells, ID, developmental delay, abnormal electroencephalography (EEG), and epilepsy have also been documented in affected individuals [4,16,17]. Notably, rare reports of speech delay have also been reported in a few cases [4,15,17].

According to a gene ontology study, apoptosis, cell proliferation, Pol II-associated transcription, and Notch signaling pathway genes are enriched in *MED23*-deficient neural stem cells. These findings show that *MED23* is a key regulator of adult brain activity when taken as a whole [18]. The clinical phenotype of the condition has not yet been thoroughly elucidated owing to the small number of reported patients with biallelic *MED23* mutations [19]. In recent years, substantial advancements were made with the emergence of new diagnostic modalities, including next-generation sequencing, which became the core technology for gene discovery, giving practicing clinicians the ability to detect novel mutations, including *MED23* [20]. In such patients, genetic testing, mainly whole exome sequencing (WES), is the modality of choice to detect the mutation, as demonstrated in previous reports.

The aim of this study was to provide further evidence to the currently available literature on the clinical presentation of the *MED23* mutation and systematically analyze the common features that could potentially be described as a syndrome, to aid clinicians in identifying patients with the mutation by emphasizing specific hallmarks related to it. Moreover, we present a novel case, the first to be reported from Saudi Arabia, carrying a mutation in the *MED23* gene, clinically presenting with seizure and developmental delay and diagnosed using advanced genetic testing.

## MATERIAL AND METHODS

### Search strategy

We reviewed the literature searching for patients with confirmed variant in the *MED23* gene. All publications, from the first published article in February 1999 to September 2023 were searched, collected, and analyzed accordingly. The terms (*MED23*) and (Mediator complex) were applied, and articles were filtered without restriction on the study design. The review was carried out in September 2023 and involved the collection and analysis of demographic, clinical, genetical, neuroimaging, and electrophysiological data. The data were collected from 16 patients [3,4,15–17,19–21] in addition to a novel case presented from Saudi Arabia. All cases had a confirmed variant in the *MED23* gene. The following databases were used for the literature search: MEDLINE/PubMed, Google Scholar, EMBASE, Scopus, Web of Science, and EBSCOhost. The extracted variables included clinical data such as seizure semiology, ID, developmental milestones, and current or past history of hypotonia. Seizure types and electroclinical syndromes were classified according to the International League Against Epilepsy (ILAE) [22]. Also, findings on neuroimaging and electrophysiological including magnetic resonance imaging (MRI) and EEG were obtained. We also extracted demographic and genetic information of each case, including age of disease onset/diagnosis, sex, reported country, parents’ origin, consanguinity, genetic mutation (i.e., patient allele, paternal allele, and maternal allele). The search strategy was constructed from previously published literature [23]. The study adhered to the provisions of the Narrative Review Checklist developed by Green *et al*. in 2006 [24]. The novel case was presented in accordance to the Case Report (CARE) guidelines for case reports [25].

### Sample collections

Following appropriate ethical and logistical measures, we obtained an informed consent from the parents of the affected child after explaining the nature and purpose of the study. Subsequently, the genetic sample of the patient was obtained.

WES testing was performed on DNA extracted from the patient’s blood, saliva, or tissue. Approximately 45 Mb of the genome, corresponding to 99% of the Consensus Coding Sequence (CCDS) obtained from RefSeq, GENCODE, ENSEMBL, were enriched from fragmented DNA with probes designed against the human genome (Nextera DNA Flex Pre-Enrichment Library Prep and Illumina Exome Panel). The library generated was sequenced with S2/S4 Reagents Kits (Illumina) on the NovaSeq 6000 Sequencing System (Illumina). Raw sequencing data were processed by the Igenomix in-house bioinformatics pipeline (v.1.0). In brief, the raw data was first demultiplexed to link molecular barcodes with the sample identification, followed by the trimming of adaptors. The reads were mapped to the human genome reference (GRCh37), and duplicated reads were marked before variant calling and annotation.

The bioinformatics procedure includes the detection of germline single nucleotide variants (SNV), small insertions or deletions, and copy number variations (CNVs). The whole exome used has an average reading depth greater than 100×, and 95% of the regions have a reading depth greater than 20×. Samples that do not meet the quality criteria established in the validation plan are evaluated in order to identify the cause of the failure and to request resampling, re-extraction, or resequencing of the sample, whenever applicable. Data analysis, including alignment to the hg19 human reference genome (Genome Reference Consortium GRCh37), variant calling, and annotation was performed using validated software [21]. The Picard tool (version 1.118) was used to remove PCR duplications. Further prioritization was performed focusing on rare variants that were loss of function (frameshift, nonsense, and splice site mutations), homozygous missense and/or affecting known disease genes from the Online Mendelian Inheritance in Man database [26].

Several prediction tools were used to predict the pathogenicity of the identified variant using in silico pathogenicity prediction programs (SIFT, Polyphen, Mutation Taster, CADD, etc). All variants related to the phenotype of the patient, except benign or likely benign variants, are reported. Additionally, provided family history and clinical information are used to evaluate eventually identified variants.

## RESULTS

### Case presentation

**Figure 1:**
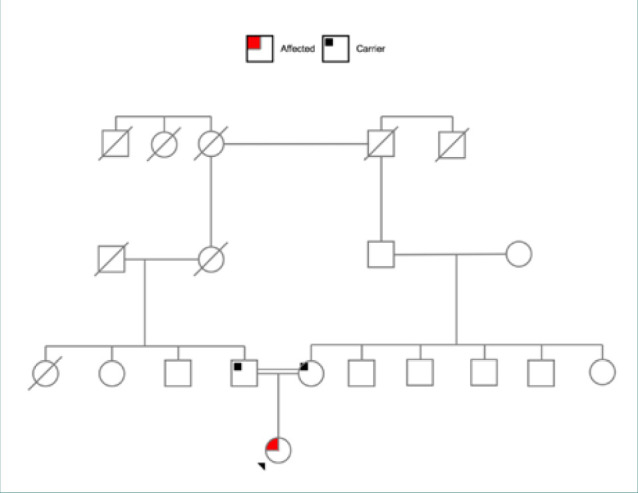
The pedigree of the family and the affected proband

The parents of the patient provided written consent for this case report. The patient was a 5-year-old Egyptian girl who presented with multiple complaints. The patient has a positive history of constipation, frequent aberrant movements resembling chorea, hypotonia, focal right-side clonic seizures, and global developmental delay. Owing to the patient’s clinical presentation and family history, she underwent WES testing, which revealed a homozygous variant in the *MED23* gene (*MED23*:c.3742G>A[p.Glu1248Lys]), a variant of uncertain significance. The patient’s parents are also of Egyptian origin and distant cousins (consanguineous marriage). The family pedigree is presented in Figure 1. She is their first child, and there are no similar health issues reported in the paternal and maternal family.

As for her perinatal history, she was born through natural vaginal birth at 39 weeks of gestation. She had normal birth parameters with a weight of 3.8 kg and a length of 56.8 cm. Initially, she attended our pediatric neurology clinic for her seizure and developmental delay. The parents were mainly concerned about their child’s development and noticed a delay in comparison to children of her age when she was 6 months of age. Currently, she is 5 years old. As for her current developmental status, she is unable to sit, stand, or walk. Rolling over is also not achieved, and she has difficulty reaching objects. In terms of speech, her abilities are limited to babbling. Cognitive function is characterized by pursuit fixation and following objects. Past medical history reveals no recurrent infections. Chronic constipation is present, requiring the patient to use laxatives. The patient was diagnosed with cerebral palsy at the age of 1 year, before the genetic testing. Physical examination findings revealed microcephaly, as head circumference was below the 3rd percentile, axial hypotonia, and appendicular spasticity. The cranial nerve examination was normal. She had no dysmorphic features and her motor function was three out of five, as the patient was able to move against gravity in a random manner. Deep tendon reflexes are normal, and sensory response includes withdrawal from painful stimuli. Choreoathetosis movements are also noted. Examining other systems was unremarkable. Furthermore, she underwent an EEG that showed left centro-temporal epileptic discharges. Brain MRI showed diffuse brain atrophy and a dilated lateral ventricle with a small right hippocampus. The patient continues to follow-up with our clinic for seizure control evaluation.

### Regulatory processes

There were 22 studies presenting novel theories of *MED23* expression pattern [9–11,14,18,27–43]. Regulatory processes are multisystemic and involve multiple molecular pathogeneses. In chronological order, ranging from most recent to oldest, the regulatory processes included the gene’s involvement in Alzheimer’s disease by serving as a co-expressive link, embryonic lethality by disturbing neural development, namely learning and memory functions, and promoting angiogenesis, as well as maintaining vascular integrity by suppressing Ang2 signaling. In vivo trials showed that alteration in the gene is involved in multiple carcinogenic activities. Furthermore, the gene inhibits tumorigenicity of esophageal squamous cell carcinoma, tumorigenicity of lung cancer cells, and tumorigenesis in hepatocellular carcinoma. It also serves as a co-expression module for gastric cancer and is a potential suppressor of colorectal cancer, as well as a potential co-predictor of breast cancer genes. In further details, the *MED23* subunit restricts smooth muscle cell lineage development and promotes growth-related gene expression. As for the gene’s role in the human brain, another study reported a regulatory mechanism in the brain’s neurogenesis and functions. In the study by Chen *et al*. [18], *MED23*-deficient mice were shown to have a decreased activity in the neuroblasts and immature neurons. Overall, other processes were summarized in Table 1, showing inconsistent findings across the human body with different involvement in multiple organs. But generally, a growing trend towards the discovery of carcinogenic involvement is noted.

**Table 1:** Main regulatory processes for the *MED23* gene

Authors	Year	Regulatory process/outcome
Ribeiro-Dos-Santos *et al*. [38]	2023	Co-expression modules for Alzheimer’s disease
Yang *et al*. [37]	2023	Embryonic lethality and disturbed neural development, learning and memory functions
Yang *et al*. [36]	2022	Promotes angiogenesis and maintains vascular integrity by suppressing Ang2 signaling
Sun *et al*. [35]	2021	Regulates smooth muscle development
Dash *et al*. [34]	2021	Involved in craniofacial anomalies
Morais-Rodrigues *et al.* [33]	2020	Potentially co-predictor of breast cancer genes
Chen *et al*. [18]	2020	Regulates adult brain neurogenesis and functions
Wang *et al*. [32]	2019	Involved in the development of experimental liver fibrosis
Xu *et al*. [31]	2018	Transcriptional regulator that controls invariant natural killer T cell differentiation and terminal maturation
Chen *et al*. [30]	2018	Gatekeeper of myeloid potential of hematopoietic stem cells
Xia *et al*. [29]	2017	Coupling ultraviolet-induced DNA repair to pigmentation
Liu *et al*. [28]	2016	Involved in a regulatory network of anabolic bone formation and related diseases
Jo *et al*. [27]	2015	Potential suppressor of colorectal cancer
Guo *et al*. [43]	2015	Suppressing proliferation and tumorigenesis in hepatocellular carcinoma
Shi *et al*. [42]	2014	Suppressive role against esophageal cancer (inhibits tumorigenicity of esophageal squamous cell carcinoma)
Sun *et al*. [9]	2014	Contributes to controlling T cell activation at the transcriptional level and prevents the development of autoimmune disorders
Chu *et al*. [11]	2014	Regulator for energy homeostasis and maintains hepatic gluconeogenesis and blood glucose levels
Majewski *et al*. [41]	2013	Co-expression modules for gastric cancer
Yin *et al*. [14]	2012	Regulates cell fate decisions, cell proliferation, and migration
Yang *et al*. [10]	2012	Inhibits the proliferation and tumorigenicity of lung cancer cells with hyperactive Ras activity
Wang *et al*. [40]	2009	Links transducing insulin signaling to the transcriptional cascade during adipocyte differentiation (involved in adipogenesis)
Balamotis *et al*. [39]	2009	Embryonic lethality with defects in neural and cardiovascular systems

### Literature review

**Figure 2:**
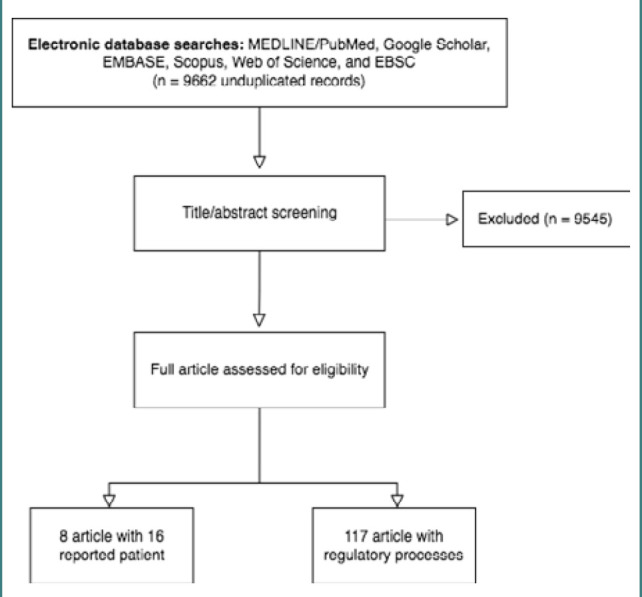
Flow diagram of the eligible and included studies

Extensive literature screening yielded 16 patients in eight published articles [3,4,15–17,19–21]. Initially, a total of 9,662 articles were filtered and screened for patients with confirmed *MED23* variant. Of those articles, 117 were eligible for further screening using the full text, whereas others were excluded using the title and abstract information. Finally, the 16 patients represent every reported case in the literature (Figure 2). All 16 patients had a confirmed variant in the *MED23* gene using advanced genetic testing. The demographic, clinical, neuroimaging, and electrophysiological data of all cases were extracted to compare findings with a novel case identified using WES testing, which has never been described in the literature.

### Exome sequencing

WES analysis was used to identify the variant in the *MED23* gene of our case. An autosomal recessive homozygous missense variant in the exon 28/31 of the *MED23* gene [NM_015979.4:c.3742G>A, p.(Glu1248Lys)] that replaces the amino acid glutamine with lysine at codon 1248 was detected. Of the 16 cases, only eight pathogenic variants were identified including *MED23*: c.383G>A(p.Gly128Glu)/c.1831C>T(p.Arg611Trp), c.2549T>C(p.Leu850Pro), c.382G>A(p.G128R)/c.539C>A(p.A180D), c.670C>G:p.R224G/c.670C>G(p.R224G), and c.1937A>G(p.Gln646Arg). Those variants were detected in only a single patient. Meanwhile, other genetic variants, including c.506A>G(p.Y169C)/c.506A>G(p.Y169C), c.3638A>G(p.H1213R)/c.3988C>T(p.R1330), and c.1832G>A(p.R611Q)/c.1832G>A(p.R611Q) were reported in multiple siblings in a single family each. Of those patients, only a single patient ascertained homozygous mutation in a novel missense variant; other patients were heterozygous mutations. Detailed genetic data in respect to their demographic data, including the patients’ and parents’ origin or ethnicity, are presented in Table 2.

**Table 2:** Patients’ demographic, genetic, and clinical data

Patient no.	Authors	Age of onset	Sex	Reported country	Parents’ origin	Consanguinity	Zygosity	Patient allele	Facial dysmorphism	Seizures	Intellectual disability	Developmental delay	Hypotonia	EEG findings	MRI findings
1	Our patient	6 m	F	Saudi Arabia	Saudi	+	Hom	*MED23*:c.3742G>A(p.Glu1248Lys)	−	+	−	+	+	+	+
2	Salzano *et al.*, 2023	At birth	F	Italy	NM	−	Het	*MED23*:c.383G>A(p.Gly128Glu)/c.1831C>T(p.Arg611Trp)	+	+	+	+	+	+	+
3	Ignatius *et al.*, 2020	18 m	F	Finland	NM	NM	Het	*MED23*:c.2549T>C(p.Leu850Pro)	−	−	−	−	+	NM	−
4	Demos *et al.*, 2019	51.5 m	M	Canada	NM	−	Het	*MED23*:c.382G>A(p.G128R)/c.539C>A(p.A180D)	−	+	+	+	−	+	−
5	Hashemi-Gorji *et al.*, 2019	Infancy	M	Iran	NM	+	Hom	MED23:c.670C>G(p.R224G)/c.670C>G(p.R224G)	−	−	+	+	+	NM	NM
6	Riazuddin *et al.*, 2017	NM	M	Pakistan	Pakistani	+	NM	*MED23*:c.506A>G(p.Y169C)/c.506A>G:(p.Y169C)	−	−	+	−	−	NM	NM
7	Riazuddin *et al.*, 2017	NM	M	Pakistan	Pakistani	+	NM	*MED23*:c.506A>G(p.Y169C)/c.506A>G:(p.Y169C)	−	−	+	−	−	NM	NM
8	Riazuddin *et al.*, 2017	NM	M	Pakistan	Pakistani	+	NM	*MED23*:c.506A>G(p.Y169C)/c.506A>G:(p.Y169C)	−	−	+	−	−	NM	NM
9	Riazuddin *et al.*, 2017	NM	F	Pakistan	Pakistani	+	NM	*MED23*:c.506A>G(p.Y169C)/c.506A>G:(p.Y169C)	−	−	+	−	−	NM	NM
10	Lionel *et al.*, 2016	5 m	M	Canada	NM	+	NM	*MED23*:c.1937A>G(p.Gln646Arg)	+	+	−	+	+	+	+
11	Trehan *et al.*, 2015	12 m	M	U.S.	Hungarian/ Czechoslovakian/ Polish	−	NM	*MED23*:c.3638A>G(p.H1213R)/c.3988C>T(p.R1330)	−	−	+	+	+	+	+
12	Trehan *et al.*, 2015	22 m	M	U.S.	NM	−	NM	*MED23*:c.3638A>G(p.H1213R)/c.3988C>T(p.R1330)	−	−	+	+	+	+	+
13	Hashimoto *et al.*, 2011	NM	F	France	Algerian	+	NM	*MED23*:c.1832G>A(p.R611Q)/c.1832G>A(p.R611Q)	−	−	+	+	−	−	−
14	Hashimoto *et al.*, 2011	NM	F	France	Algerian	+	NM	*MED23*:c.1832G>A(p.R611Q)/*MED23*:c.1832G>A(p.R611Q)	−	−	+	+	−	−	−
15	Hashimoto *et al.*, 2011	NM	F	U.S.	Algerian	+	NM	*MED23*:c.1832G>A(p.R611Q)/*MED23*:c.1832G>A(p.R611Q)	NM	NM	+	NM	NM	NM	NM
16	Hashimoto *et al.*, 2011	NM	F	U.S.	Algerian	+	NM	*MED23*:c.1832G>A(p.R611Q)/*MED23*:c.1832G>A(p.R611Q)	NM	NM	+	NM	NM	NM	NM
17	Hashimoto *et al.*, 2011	NM	M	U.S.	Algerian	+	NM	*MED23*:c.1832G>A(p.R611Q)/*MED23*:c.1832G>A(p.R611Q)	NM	NM	+	NM	NM	NM	NM

F, female; Het, heterozygous; Hom, homozygous; m, months; M, male; NM, not mentioned; U.S., United States. + indicates the presence of the variable, and − indicates the absence of the variable

### Baseline clinical data

**Figure 3:**
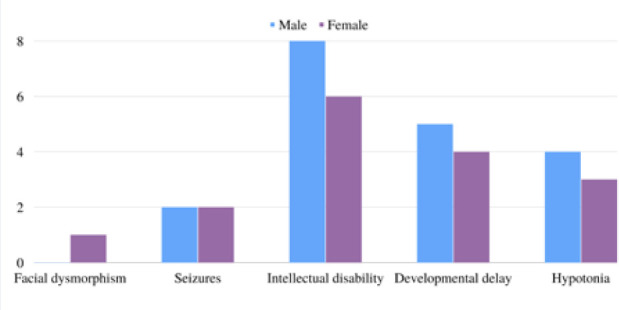
The prevalence of main clinical features compared according to sex distribution

Key baseline data include the age of onset that was reported among eight patients. The age of disease onset ranged from since birth (patient no. 1) to 51.5 months (patient no. 4). Regarding clinical features, facial dysmorphism was reported in two patients (patient no. 2 and no. 10). Seizures were reported in three patients in addition to our patient. Their semiology included generalized tonic seizures across all three patients, with one presenting with tonic-clonic seizures. Regarding our patient, she presented with focal right-side clonic seizures. Moreover, ID was the most prevalent manifestation, presenting in 14 patients, two of which reported positive for seizure activity. Developmental delay with various degrees of severity and across a spectrum of domains was also present in nine patients. Lastly, hypotonia was reported among seven patients. Detailed data are presented in Table 2. A summary of the clinical presentation is illustrated in Figure 3.

### Neuroimaging and electrophysiological data

Regarding EEG studies, abnormal findings were detected in five patients. Similarly, another five patients did not perform EEG studies. Normal EEG findings were reported among two patients. Both patients with normal EEG clinically reported ID and developmental delay. Despite abnormal EEG in patients no. 11 and 12, there were no clear seizure or epileptic activity clinically. Abnormal EEG included slow backgrounds and spike and sharp-wave complexes. Concerning neuroradiological findings, abnormal MRI was reported among four patients, and their findings included delayed myelination, thin corpus callosum, and pontine hypoplasia. Detailed radio-electrophysiological data are presented in Table 2.

## DISCUSSION

*MED23* is a gene encoding a subunit of the mediator complex that integrates various signaling pathways [41,42]. It has multiple molecular roles, including an oncogenic role in Ras-dependent cancers [10]. In some overexpressed processes, silencing *MED23* promotes the role of adjuvant therapies that could contribute to overcoming drug resistance in some patients [43]. Knockout mice studies showed multiple involvement of the gene in transcription factors, possibly potentiating the expression of other factors [41]. This gene has shown involvement in adipogenesis, chromatin modification, neural differentiation, proliferation, smooth muscle cell differentiation, and tumorigenesis [3,34,44,45].

**Figure 4:**
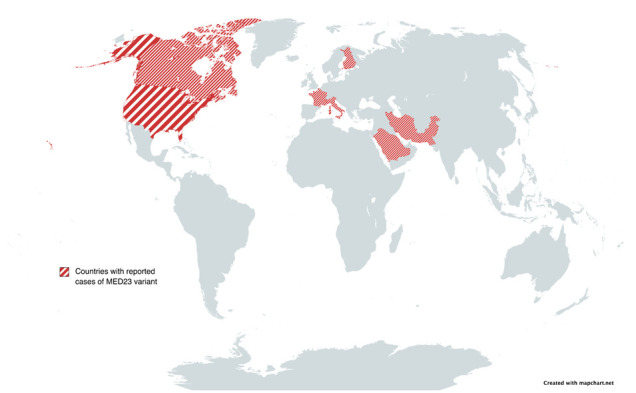
Countries with reported cases of *MED23* mutation (Saudi Arabia, Iran, Pakistan, United States, Canada, France, Italy, and Finland)

In this study, we summarized all regulatory processes of the gene, indicating a broad spectrum of involvement. *MED23* interacts with several transcription activators involved in splicing, elongation, and post-transcriptional events [46]. Moreover, many studies reported different phenotypic features of the patients carrying a pathogenic variant of the gene in limited countries across the globe (Figure 4) [4,15,17,19]. To name a few, some studies reported facial dysmorphism among their patients [17,19], despite the lack of such findings among other patients [3,4,16,39,40] in addition to our newly-reported case. Even in some families with multiple affected individuals, such findings were absent [40]. Furthermore, seizure was not a predominant hallmark, with only three cases in the literature reporting some form of seizure activity [16,17,19]. However, although our case presented with seizure, the literature suggests that it is less expected that the *MED23* gene would be considered a part of genetic epilepsy disorders. In one case reported by Lionel *et al*. [17], a ketogenic diet was effective in controlling the patient’s seizure.

As far as ID is concerned, it is a potential hallmark for the condition, with the majority of patients reporting a positive finding in this area. The association between *MED23* and ID was first proposed by Hashimoto *et al*. in 2011 [3] in his non-syndromic family that included five affected siblings using homozygosity mapping and linkage analysis. Since then, other cases have been reported with similar findings, despite carrying different variants [4,15,16,19,40]. In the current study, our patient manifested clinical symptoms similar to previously described cases, mainly seizure, developmental delay, hypotonia, and microcephaly. However, we did not report any findings that have not been previously described. Moreover, our patient presented with abnormal EEG and MRI findings, which was consistent with the majority of the reported cases, highlighting another potential hallmark of the condition. Nonetheless, even with the growing number of cases and efforts that have been made to summarize and illustrate a possible common phenotype for the condition, the clinical and electrophysiological presentation of *MED23* remains variable, raising difficulties in suspecting such variant in real-life practice. However, genetic testing, mainly WES, showed efficacy in overcoming such difficulties and is convincing in detecting this rare pathogenic variant. There has been a lack of studies that track the evolution of this condition over the past years; however, this gap is addressed by this study, which can serve as a track record for future studies.

## CONCLUSION

Considerable progress has been made in the study of the structure and function of the *MED23* gene in recent years. So far, many case reports associated the gene with a broad spectrum of phenotypic features with neurodegenerative nature. On a molecular scale, the gene was demonstrated to involve multiple processes with self-renewal and cell cycle progression activity. In this study, we provided insight into the collective phenotype of the disorder by collecting and analyzing all the reported cases. On a molecular level, many studies emphasized different involvement of the gene in multisystemic regulatory processes. After reporting an additional novel case, we highlight ID, developmental delay, hypotonia, and microcephaly as potential clinical hallmarks of *MED23* mutations owing to their repetitive appearance among diagnosed cases. Abnormal EEG and MRI findings can also be considered as supporting hallmarks, which can also aid in suspecting such cases before using genetic testing. Whenever a clinical suspicion arises, we emphasize the importance of genetic testing, particularly WES, in definitively detecting the mutation. Further studies are needed to track the evolution of this condition and aid in its early identification, potentially leading to its classification as a syndrome. The data we presented provide a strong basis for future investigations about the possible roles of *MED23* in the development and function of the human body, as well as understanding its clinical phenotype.

## Data Availability

Further data are available from the corresponding author upon reasonable request.

## References

[1] Hwa JB, Yun KK, Roeder RG (2006). Human Mediator enhances basal transcription by facilitating recruitment of transcription factor IIB during preinitiation complex assembly. J Biol Chem.

[2] Ji X, Fu XD (2012). The Mediator couples transcription and splicing. Mol Cell.

[3] Hashimoto S, Boissel S, Zarhrate M, Rio M, Munnich A, Egly JM (2011). *MED23* mutation links intellectual disability to dysregulation of immediate early gene expression. Science.

[4] Trehan A, Brady JM, Maduro V, Bone WP, Huang Y, Golas GA (2015). *MED23*associated intellectual disability in a non-consanguineous family. Am J Med Genet Part A.

[5] Hentges KE (2011). Mediator complex proteins are required for diverse developmental processes. Semin Cell Dev Biol.

[6] Bourbon HM, Aguilera A, Ansari AZ (2004). A unified nomenclature for protein subunits of mediator complexes linking transcriptional regulators to RNA polymerase II. Mol Cell.

[7] Malik S, Roeder RG (2010). The metazoan Mediator co-activator complex as an integrative hub for transcriptional regulation. Nat Rev Genet.

[8] Zhao C, Deng W, Gage FH (2008). Mechanisms and functional implications of adult neurogenesis. Cell.

[9] Sun Y, Zhu X, Chen X (2014). The mediator subunit *Med23* contributes to controlling T-cell activation and prevents autoimmunity. Nat Commun.

[10] Yang X, Zhao M, Xia M, Liu Y, Yan J, Ji H, Wang G (2012). Selective requirement for Mediator *MED23* in Ras-active lung cancer. Proc Natl Acad Sci U S A.

[11] Chu Y, Rosso LG, Huang P (2014). Liver *Med23* ablation improves glucose and lipid metabolism through modulating FOXO1 activity. Cell Res.

[12] Liu Z, Yao X, Yan G, Xu Y, Yan J, Zou W, Wang G (2016). Mediator *MED23* cooperates with RUNX2 to drive osteoblast differentiation and bone development. Nat Commun.

[13] Yao X, Tang Z, Fu X (2015). The Mediator subunit *MED23* couples H2B mono-ubiquitination to transcriptional control and cell fate determination. EMBO J.

[14] Yin J wen, Liang Y, Park JY (2012). Mediator *MED23* plays opposing roles in directing smooth muscle cell and adipocyte differentiation. Genes Dev.

[15] Hashemi-Gorji F, Fardaei M, Tabei SMB, Miryounesi M (2019). Novel mutation in the *MED23* gene for intellectual disability: A case report and literature review. Clin Case Reports.

[16] Demos M, Guella I, DeGuzman C (2019). Diagnostic Yield and Treatment Impact of Targeted Exome Sequencing in Early-Onset Epilepsy. Front Neurol.

[17] Lionel AC, Monfared N, Scherer SW, Marshall CR, Mercimek-Mahmutoglu S (2016). *MED23*associated refractory epilepsy successfully treated with the ketogenic diet. Am J Med Genet A.

[18] Chen GY, Zhang S, Li CH (2020). Mediator *Med23* Regulates Adult Hippocampal Neurogenesis. Front cell Dev Biol.

[19] Salzano E, Niceta M, Pizzi S (2023). Case report: Novel compound heterozygosity for pathogenic variants in *MED23* in a syndromic patient with postnatal microcephaly. Front Neurol.

[20] Ignatius E, Isohanni P, Pohjanpelto M (2020). Genetic background of ataxia in children younger than 5 years in Finland. Neurol Genet.

[21] Riazuddin S, Hussain M, Razzaq A (2017). Exome sequencing of Pakistani consanguineous families identifies 30 novel candidate genes for recessive intellectual disability. Mol Psychiatry.

[22] Fisher RS, Cross JH, French JA (2017). Operational classification of seizure types by the International League Against Epilepsy: Position Paper of the ILAE Commission for Classification and Terminology. Epilepsia.

[23] Muthaffar OY, Jan MMS, Alyazidi AS, Alotibi TK, Alsulami EA (2023). Insight into Genetic Mutations of SZT2: Is It a Syndrome?. Biomedicines.

[24] Green BN, Johnson CD, Adams A (2006). Writing narrative literature reviews for peer-reviewed journals: secrets of the trade. J Chiropr Med.

[25] Gagnier JJ, Kienle G, Altman DG (2013). The CARE guidelines: Consensus-based clinical case reporting guideline development. J Med Case Rep.

[26] Tammimies K, Marshall CR, Walker S (2015). Molecular Diagnostic Yield of Chromosomal Microarray Analysis and Whole-Exome Sequencing in Children With Autism Spectrum Disorder. JAMA.

[27] Jo YS, Kim MS, Lee SH, Yoo NJ (2015). Mutational Heterogeneity of *MED23* Gene in Colorectal Cancers. Pathol Oncol Res.

[28] Liu Z, Yao X, Yan G, Xu Y, Yan J, Zou W, Wang G (2016). Mediator *MED23* cooperates with RUNX2 to drive osteoblast differentiation and bone development. Nat Commun.

[29] Xia M, Chen K, Yao X (2017). Mediator *MED23* Links Pigmentation and DNA Repair through the Transcription Factor MITF. Cell Rep.

[30] Chen X, Zhao J, Gu C (2018). *Med23* serves as a gatekeeper of the myeloid potential of hematopoietic stem cells. Nat Commun.

[31] Xu Y, Sun Y, Shen H (2018). Regulation of the terminal maturation of iNKT cells by mediator complex subunit 23. Nat Commun.

[32] Wang Z, Cao D, Li C, Min L, Wang G (2019). Mediator *MED23* regulates inflammatory responses and liver fibrosis. PLoS Biol.

[33] Morais-Rodrigues F, Silv́erio-Machado R, Kato RB (2020). Analysis of the microarray gene expression for breast cancer progression after the application modified logistic regression. Gene.

[34] Dash S, Bhatt S, Falcon KT, Sandell LL, Trainor PA (2021). *Med23* Regulates Sox9 Expression during Craniofacial Development. J Dent Res.

[35] Sun X, Yin J wen, Liang Y, Li C, Gao P, Yu Y (2021). Mediator *Med23* deficiency in smooth muscle cells prevents neointima formation after arterial injury. Cell Discov.

[36] Yang Y, Xiao Q, Yin J (2022). *Med23* supports angiogenesis and maintains vascular integrity through negative regulation of angiopoietin2 expression. Commun Biol.

[37] Yang Y, Li C, Chen Z (2023). An intellectual disability-related *MED23* mutation dysregulates gene expression by altering chromatin conformation and enhancer activities. Nucleic Acids Res.

[38] Ribeiro-dos-Santos A, de Brito LM, de Araújo GS (2023). The fusiform gyrus exhibits differential gene-gene co-expression in Alzheimer’s disease. Front Aging Neurosci.

[39] Balamotis MA, Pennella MA, Stevens JL, Wasylyk B, Belmont AS, Berk AJ (2009). Complexity in transcription control at the activation domain-mediator interface. Sci Signal.

[40] Wang W, Huang L, Huang Y, Yin J wen, Berk AJ, Friedman JM (2009). Mediator *MED23* links insulin signaling to the adipogenesis transcription cascade. Dev Cell.

[41] Majewski IJ, Kluijt I, Cats A (2013). An α-E-catenin (CTNNA1) mutation in hereditary diffuse gastric cancer. J Pathol.

[42] Shi J, Han Q, Zhao H, Zhong C, Yao F (2014). Downregulation of *MED23* promoted the tumorigenecity of esophageal squamous cell carcinoma. Mol Carcinog.

[43] Guo Y, Wang J, Li H (2015). Mediator subunit 23 overexpression as a novel target for suppressing proliferation and tumorigenesis in hepatocellular carcinoma. J Gastroenterol Hepatol.

[44] Kasper LH, Fukuyama T, Brindle PK (2014). T-cells null for the *MED23* subunit of mediator express decreased levels of KLF2 and inefficiently populate the peripheral lymphoid organs. PLoS One.

[45] Stevens JL, Cantin GT, Wang G, Shevchenko A, Shevchenko A, Berk AJ (2002). Transcription control by E1A and MAP kinase pathway via Sur2 mediator subunit. Science.

[46] Zhu W, Yao X, Liang Y (2015). Mediator *Med23* deficiency enhances neural differentiation of murine embryonic stem cells through modulating BMP signaling. Development.

